# Health of New York

**Published:** 1866-05

**Authors:** 


### HEALTH OF NEW YORK.

The city of New York is capable of being made the healthiest of all the large cities of the world, and is one of the healthiest now. Much has been said about the filthiness of its streets, its underground habitations, and its crowded tenement houses, but unfortunately, the speakers and writers have not been disinterested persons, or if so, were careless in their statements, if not very ignorant of that about which they were writing. When the Hub of the Universe wishes to compare favorably against New York as to health, she gives the population and the deaths of each city, knowing at the same time that the foundation is false, for New York gives the deaths from all causes, and the regulations are so stringent that no dead body can be conveyed from the city or be buried, without official permission and faithful registry; but in Boston, the still-born are not counted, in New York they are. From time immemorial, Philadelphia stoutly has contended, and still believes, that she is larger than New York, the celebrated Frog entertained a similar opinion as to the ox, but exploded in attempting to figure it out; she claims that she has more houses than Gotham, and that the only reason why New York has a greater number of inhabitants set down to her is that the population is counted twice, because the people live on one end of the island and do business on the other, and when the census is taken, the wives are called upon at their residences, and the husbands are called upon at their places of business, thus making the returns just double.

But when Uncle Penn wants to prove that the right-angled city, with wet pavements and white door steps and green window shutters, is incomparably more healthy than the great metropolis, she believes the population statistics are most religiously true. So with the penny-a-liners and speech-makers, who have axes to grind; they compare the total number of deaths annually, with the totals given of other large cities, knowing at the time, or blissfully ignorant, that New York gives all the deaths, when some ought not, in justice, to be counted, and are not counted elsewhere. While Boston does not count the still-born, Liverpool does not count those who die in the city who have lately come from the country, and this really ought not to be the case in endeavoring to arrive at the healthfulness of any locality. The Liverpool Registrar did not count the Irish deaths for 1847—that would have run up the death rate three per cent. making it 39 per 1000, instead of 36. So few foreigners go to Paris, that only about two hundred die there in a year, while as to New York, out of every five deaths four are foreign. More foreigners land in New York city in a month, than at Philadelphia and Boston both together in a year. In 1865, one hundred and eighty-three thousand emigrants landed in NewYork city: in three days in July, thirty-two emigrants died, more than ten a day, some of them dying twenty-four hours after their arrival, and in nearly all the cases, the deaths were the result of sickness of long duration, acquired abroad. Leaving out of the account, the still-born, and those of emigrants who die on landing, New York city would give a death-rate as favorable, perhaps, as any large seaport on the globe. From fifteen to twenty per cent. of deaths in New York are of foreigners who contracted their diseases before they reached the city; such deaths ought not to be set down to the unhealthfulness of New York. If as between New York, Boston and Philadelphia, the still-born were excluded or admitted in the mortuary returns of each ; and the deaths of foreigners who contracted their sickness before they reached this country, were not counted, it would be seen at a glance, that New York city, as a healthful residence, has been greatly maligned. One plain indisputable fact is of very great significance, giving round numbers: while the native and foreign born population of New York is about equally divided, eleven thousand children of foreign born parents died in New York during 1864, while of children born of native parents, less than two thousand died, giving as a general fact, that five out of six of the children dying in a year in New York city, five are born of foreign parents.

